# Raman Study of Strain Relaxation from Grain Boundaries in Epitaxial Graphene Grown by Chemical Vapor Deposition on SiC

**DOI:** 10.3390/nano9030372

**Published:** 2019-03-05

**Authors:** Laiyuan Chong, Hui Guo, Yuming Zhang, Yanfei Hu, Yimen Zhang

**Affiliations:** The State Key Discipline Laboratory of Wide Band Gap Semiconductor Technology, School of Microelectronics, Xidian University, Xi’an 710071, China; chonglaiyuan@163.com (L.C.); zhangym@xidian.edu.cn (Y.Z.); ymzhang@xidian.edu.cn (Y.Z.)

**Keywords:** Raman, strain relaxation, grain boundaries, epitaxial graphene

## Abstract

Strains in graphene play a significant role in graphene-based electronics, but many aspects of the grain boundary effects on strained graphene remain unclear. Here, the relationship between grain boundary and strain property of graphene grown by chemical vapor deposition (CVD) on the C-face of SiC substrate has been investigated by Raman spectroscopy. It is shown that abundant boundary-like defects exist in the graphene film and the blue-shifted 2D-band frequency, which results from compressive strain in graphene film, shifts downward linearly as 1/L_a_ increases. Strain relaxation caused by grain boundary diffusion is considered to be the reason and the mechanism is analyzed in detail.

## 1. Introduction

The extraordinary electronic, optical and mechanical properties of pristine (strain-free and defect-free) graphene (e.g., excellent charge carrier mobility, optical transparency and mechanical strength) make it a promising material for semiconductor devices, effective Raman enhancement, and flexible and printable optoelectronics [1,2,3,4,5]. Strain can be used to dramatically modify the electronic structure and phonon dispersion of graphene and even introduce a band gap [6]. Thus, it is essential to investigate strained graphene for the realization of graphene-based electronics. In previous literatures to study strain property of graphene, the graphene samples were mainly mechanically exfoliated from highly oriented pyrolytic graphite (HOPG) and transferred onto polymer or SiO_2_ substrates [6,7]. Although exfoliated graphene offers a large (10–100 μm) and high-quality single domain, the film size is too small for wafer-scale applications. Alternatively, chemical vapor deposition (CVD) on metal substrates and sublimation of silicon atoms from silicon carbide are promising routes for producing wafer-size graphene film [8,9]. However, polycrystalline graphene exists in the films grown by these two methods and the grain size is much smaller than exfoliated graphene, which results in the inevitable formation of grain boundaries [10]. Grain boundary is a common geometrical defect, and consists of repeated pentagon and heptagon pairs, and sometimes octagons [11]. Such kinds of defects can severely weaken the mechanical strength of graphene and have been observed in graphene grown on SiC substrate by scanning tunneling microscopy (STM) study [12,13,14,15]. However, there were very few investigations focused on the relationship between grain boundary and strain property of polycrystalline graphene, and many aspects of the grain boundary effects on strained graphene remain unclear.

Both the strain and grain size in materials can be probed by XRD and Raman spectroscopy [16,17,18]. However, XRD is unsuitable for probing few-layer graphene films grown on SiC substrate because it needs constructive interference of x-rays reflected from a set of parallel atomic planes. Krishna et al. [18] have done a comparative study of the microstructural information extracted from the peaks of both Raman and XRD spectroscopy, and found that both techniques produced agreeable results for the graphite lattice strain and crystallite size, which proves the validity of deducing both strain and crystallite size from Raman spectra. So, Raman is used to analyze both strain and grain size in this paper. In Raman spectroscopy, the G-band (at about 1580 cm^−1^) and 2D-band (at about 2680 cm^−1^) are the fingerprints of pristine graphene. The G-band originates from in-plane vibration of sp^2^ carbon atoms and is a doubly degenerate (TO and LO) phonon mode at the Brillouin zone center. The 2D-band is the second order overtone of the D-band and originates from a two-phonon double resonance Raman process where momentum conservation is satisfied by two phonons with opposite wave vectors. In Raman spectra of strained graphene, the G-band and 2D-band peak frequencies shift due to phonon hardening or softening. Typically, compressive strain leads to phonon hardening (frequency upshift), while tensile strain results in phonon softening (frequency downshift). For defective graphene there will appear another two disorder-induced peaks at about 1350 cm^−1^ (D-band) and 1620 cm^−1^ (D’-band). The D-band involves an iTO phonon around the K-point and is an intervalley process, and the D’-band is an intravalley process connecting two points belonging to the same cone around K (or K’). 

In this work, Raman spectra of polycrystalline graphene, grown by CVD method on the C-face of SiC substrate (CVD-EG) using propane as the carbon source [8,19], are investigated. This method can provide precise graphene layer number control by adjusting the mass transport of the carbon precursor because the carbon atoms in graphene come from the decomposition of propane molecule [8]. The effect of grain boundaries on the strain property of CVD-EG is studied. A nonuniform crystallite size and 2D-band frequency distribution are observed and the blue-shifted 2D-band frequency shifts back with decreasing crystallite size.

## 2. Materials and Methods 

After ultrasonic cleaning by ethanol and acetone, the 2 × 2 cm^2^ semi-insulating C-face 4H-SiC substrate was loaded into a commercial horizontal CVD hot-wall reactor (Aixtron VP508). The chamber was pumped down for 3 h to reach the pressure of 1 × 10^−6^ kPa for venting oxygen. Then the temperature was raised up to 1250 °C under an argon flow of 20 L/min. After temperature stabilized, 6 SCCM of propane was added to the argon to grow graphene. The growth time was 20 minutes and the growth pressure was kept at 40 kPa. After growth, the chamber was self-cooled down to room temperature under argon ambient. 

The Raman measurements were carried out at room temperature using a HORIBA LabRAM HR800 system. The excitation laser energy was 2.41 eV (514.5 nm) and the laser spot size was about in diameter of 1 μm focused by a 100× objective lens. Backscattering configuration was applied with low power of 1 mW to avoid laser induced heating. The atomic force microscopy (AFM) measurements were carried out using a Bruker Dimension Edge system and the AFM images were obtained in tapping-mode.

## 3. Results and Discussion

**Figure 1:**
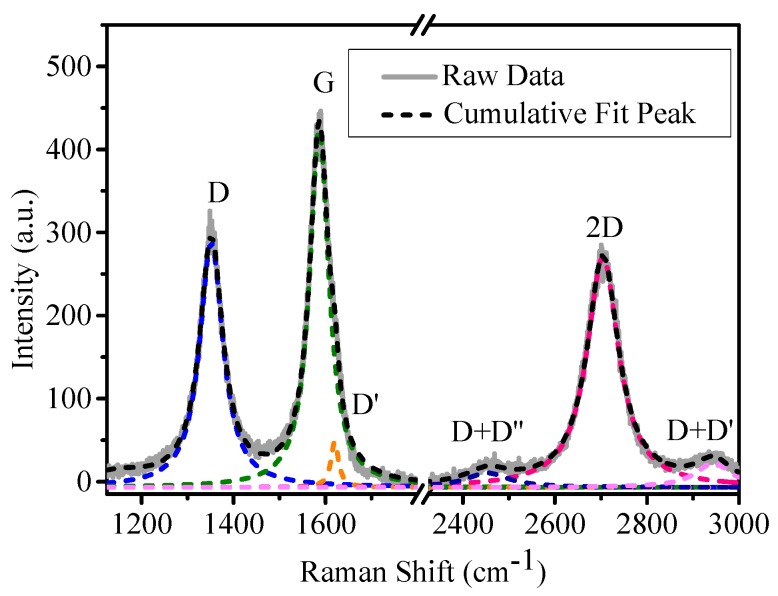
Raman spectrum and fitting peaks of polycrystalline graphene grown by chemical vapor deposition on the C-face of SiC substrate (CVD-EG).

Raman spectra were recorded at five different positions on the surface of the CVD-EG sample. Figure 1 plots the typical Raman spectrum with curve fitting. When fitting the spectrum, the contribution of the buffer layer [20,21] to the G-band is not contained because the buffer layer is not observed for graphene samples grown on the C-face of SiC substrate [22,23]. The spectrum consists of three strong peaks at 1352 cm^−1^ (D-band), 1586 cm^−1^ (G-band) and 2704 cm^−1^ (2D-band) and one weak peak at 1620 cm^−1^ (D’-band). The appearance of significant D-band and weak D’-band proves the presence of plenty of defects in the CVD-EG sample. Blue-shifts of G-band position (6 cm^−1^ shifted from 1580 cm^−1^ observed for monolayer exfoliated graphene at a laser wave-length of 514.5 nm) [24] and especially of 2D-band position (24 cm^−1^ shifted from 2680 cm^−1^) are observed, which are attributed to compressive strain in graphene layer generated during the cooling down period, because of the large thermal expansion coefficient difference between SiC and graphene [25,26].

**Figure 2:**
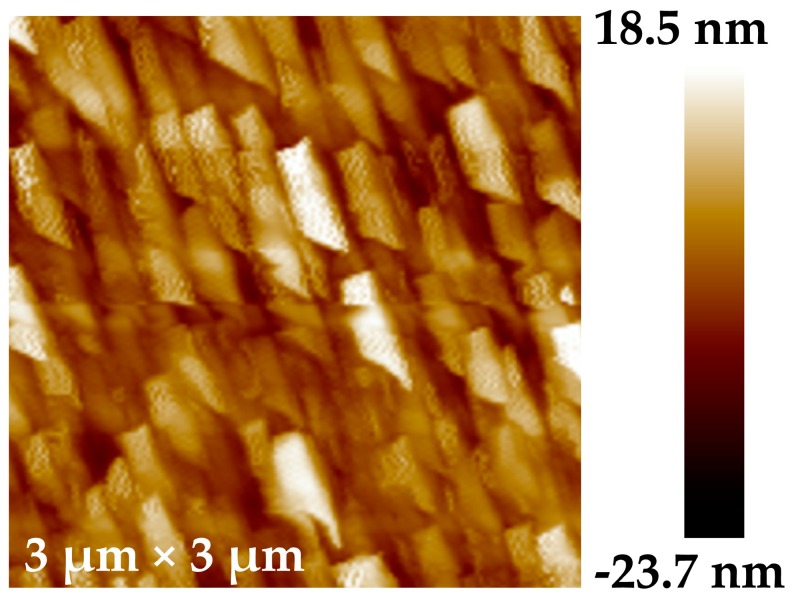
Atomic force microscopy (AFM) 3 μm × 3 μm topography image of CVD-EG.

Figure 2 presents the AFM topography image of the sample. The graphene film grows along the terraces of the SiC substrate and preserves the SiC surface morphology. Thus, the variation of terrace width in the SiC substrate leads to non-uniform grain size, which is shown in Figure 2. 

**Figure 3:**
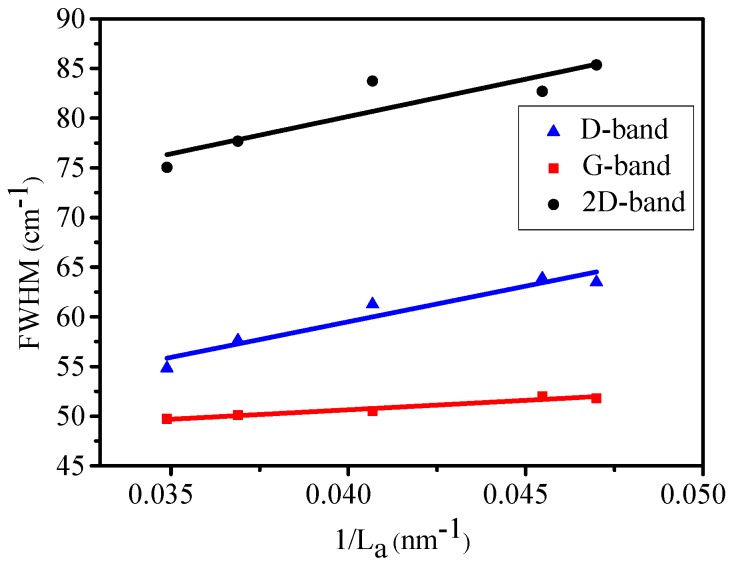
Peak widths of D, G and 2D bands as a function of 1/L_a_.

The full width at half-maximum (FWHM) of the D-band (FWHM(D)), G-band (FWHM(G)) and 2D-band (FWHM(2D)) as a function of 1/L_a_ are presented in Figure 3. L_a_ is the crystallite size of the grown graphene film, and can be calculated using the ratio of the D-band intensity (I_D_) to that of the G-band (I_G_) according to Equation (1) [18].

(1) $$L_{a}(\mathrm{nm})=2.4\times 10^{-10}\lambda^{4}{\left(\frac{I_{D}}{I_{G}}\right)}^{-1},$$

where λ is the excitation laser wavelength in nm used in the Raman measurements. The linear behavior between FWHM and 1/L_a_ can be explained as follows [27]: If the crystallite size is smaller than the phonon mean free path, the phonon lifetime τ will be proportional to the crystallite size L_a_. Since the FWHM is determined by lifetime effects in Raman bands involving resonance conditions, it can be assumed that FWHM is proportional to 1/τ and consequently FWHM is proportional to 1/L_a_. The intercept for the FWHM(2D) is 44 cm^−1^, which means FWHM(2D) has a value of 44 cm^−1^ when the crystallite size of epitaxial graphene is large. This is consistent with the FWHM(2D) of single layer graphene epitaxially grown on SiC substrate observed by Lee et al. [28]. To identify the thickness of graphene epitaxially grown on SiC substrate unambiguously, the number of Lorentzian function numbers for fitting the 2D band should be used [28,29]. A single Lorentzian fit can identify monolayer graphene, four Lorentzians are necessary for bilayers, and two Lorentzians for three-layers [30,31]. The 2D-band of all the Raman spectra can be fitted quite well by one Lorentzian peak (as shown in Figure 1), therefore, the graphene obtained in our experiment is monolayer.

Eckmann et al. [32] found that the intensity ratio of the D and D’ peak could be used experimentally to obtain the information on the type of defects in graphene, in which it is about 7:1 for vacancy-like defects and decreases to about 7:2 for the boundaries. The ratio in our sample is around 5:1, so the defects mainly consist of boundary-like defects. 

Ferrari and Robertson [33] defined a three-stage amorphization trajectory ranging from graphite to tetrahedral amorphous carbon, including graphite to nanocrystalline graphite (stage one), nanocrystalline graphite to low sp^3^ amorphous carbon (stage two) and low sp^3^ amorphous carbon to high sp^3^ amorphous carbon (stage three). The evolution of the Raman spectrum in stage one is as follows: (a) D-band appears and I_D_/I_G_ increases following Equation (1); (b) D’-band appears at about 1620 cm^−1^; (c) each FWHM for all bands is broadened due to disorder; (d) D+D’ appears. The transition between stage one and two usually occurs at I_D_/I_G_ ≈ 3 (corresponding to L_a_ ≈ 5.5 nm) using excitation laser energy of 2.41 eV [32].

**Figure 4:**
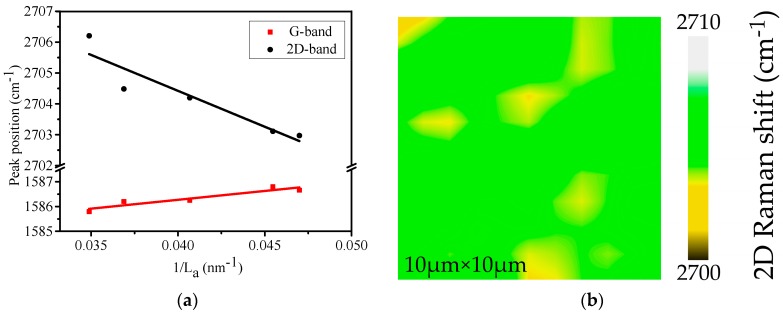
(**a**) Peak positions of G-band and 2D-band as a function of 1/L_a_, (**b**) scanning Raman map of 2D-band frequency.

L_a_ in our graphene sample is about 25 nm, and it is believed that our CVD-EG sample is in stage one, according to the three-stage amorphization trajectory and the peak characteristics shown in Figure 1 and Figure 3. Eckmann et al. [34] reported a detailed Raman study of defective exfoliated graphene and observed that the positions of G-band and 2D-band were constant in this low disorder stage. However, as shown in Figure 4a, the G-band frequency shifts upward as the crystallite size decreases, and the blue-shifted 2D-band behaves in the opposite way and shifts back with increasing defects.

Although both charge and strain could affect the positions of G and 2D bands, the unintentional electron doping of epitaxial graphene on SiC substrate was found previously to be only about 1 × 10^13^ cm^−2^ [35]. Such order of magnitude shows negligible influence on the 2D-band frequency [29,36]. Thus, the down-shift of the 2D-band frequency is attributed to the reduction of compressive strain. The mode-dependent relation between peak shift Δω and strain tensor ε is given by Equation (2) [26].

(2) $$\frac{\Delta \omega }{\omega }=-\gamma_{m}Tr\left(\varepsilon_{\mathrm{ij}}\right),$$

where γ_m_ is the mode Grüneisen parameter. For 2D-band of graphene γ_2D_ ≈ 2.7. 

Figure 4b plots the scanning Raman map of 2D-band frequency, and the homogeneous frequency distribution proves the uniformity of strain and doping across the 10 μm × 10 μm spatial region. It is noted that the five positions in Figure 4a are separated from each other by several millimeters. Thus, the big difference in 2D-band frequency between different positions and the homogeneous frequency distribution across the 10 μm × 10 μm spatial region near each position prove the robustness of the result in Figure 4a.

**Figure 5:**
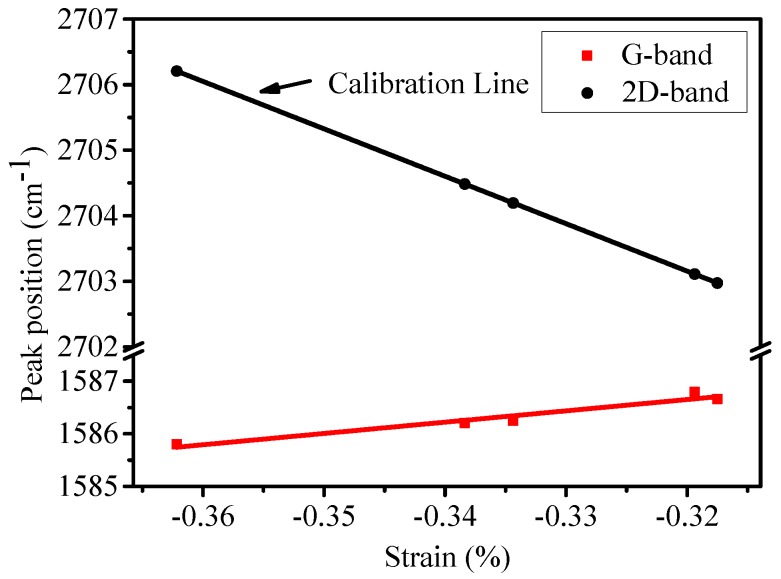
Peak positions of G-band and 2D-band as a function of strain.

Figure 5 presents the positions of G and 2D bands as a function of strain using 2D peak shift for strain calibration. It can be seen that the slope of G-band (21.5 cm^−1^/%) has opposite sign comparing with that of 2D-band (−72.4 cm^−1^/%). This phenomenon is interesting, since the lattice deforms and the G-band and 2D-band positions should shift in the same way as a consequence of phonon hardening or softening when strain occurs in single domain graphene. Bissett et al. [16] also observed the same anomalous behavior when studying the polycrystalline graphene with the crystallite size of 1 μm and they concluded that the anomalous behavior resulted from the presence of grain boundaries in graphene with small crystallite size. 

Another significant observation in Figure 4a is the linear relation between the 2D-band frequency and 1/L_a_. As mentioned above, the 2D-band keeps constant in stage one of the three-stage amorphization trajectory. So, it could not directly obtain the relation between 2D-band position and crystallite size from the effect of defects, which is attributed to the nonuniform distribution of compressive strain. In this case, the compressive strain has a larger value in the positions with larger crystallite size. This interesting property of polycrystalline graphene has never been reported before.

It is assumed that the graphene is free of strain when the growth period is over [25]. As the temperature goes down, there will be a compressive strain in the graphene film because of the much greater thermal expansion coefficient of SiC substrate than that of graphene film. Since the crystallite size is small and the type of defects is mainly boundary-like, there will be abundant grain boundaries. The atoms at the grain boundaries can diffuse in two ways [37]:

(1) lattice diffusion directly into the grains and
(2) much faster diffusion along the grain boundary.

**Figure 6:**
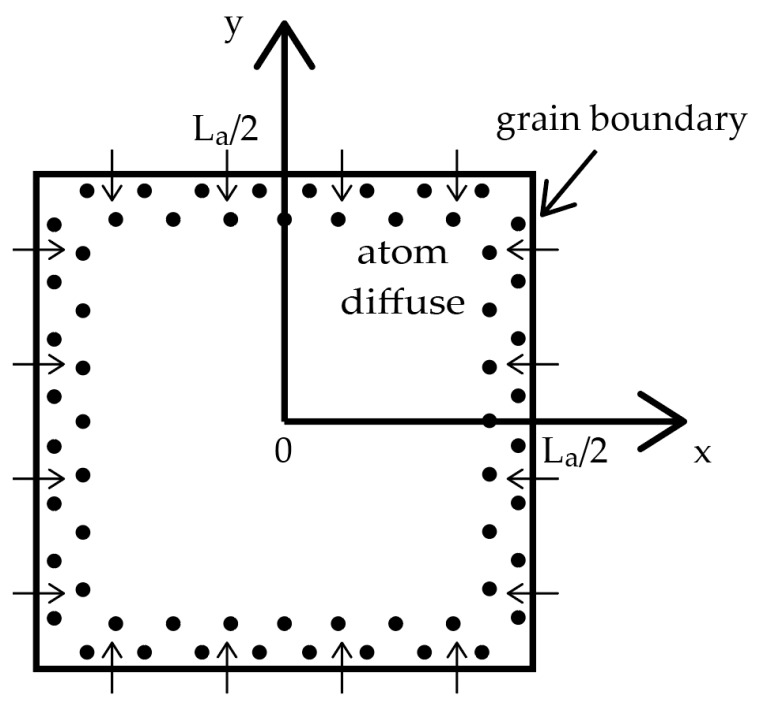
Square shape grain model of graphene.

The square shape grain model (as shown in Figure 6) is used according to the AFM image in Figure 2 and it is supposed that each grain is isolated with no mechanical interaction between neighboring grains. In steady-state diffusion, the atom distribution caused by lattice diffusion can be described by diffusion Equation (3).

(3) $$D\frac{d^{2}C\left(L_{a}/2-x\right)}{dx^{2}}=\frac{C\left(L_{a}/2-x\right)}{\tau },$$

where D and τ are diffusion coefficient and relaxation time of lattice diffusion, respectively. The solution of Equation (3) has the form of Equation (4).

(4) $$C\left(x\right)=A\exp\left(-\frac{L_{a}/2-x}{L}\right)+B\exp\left(+\frac{L_{a}/2-x}{L}\right),$$

where $L=\sqrt{D\tau }$ is the diffusion length. The boundary conditions are C(0) = 0 and C(L_a_/2) = C_0_. Here, it is assumed that the diffusion length L is much smaller than crystallite size L_a_. Thus, A = C_0_ and B = 0. The solution of Equation (3) is written by Equation (5).

(5) $$C\left(x\right)=C_{0}\exp\left(-\frac{L_{a}/2-x}{L}\right).$$

The diffusion of atoms will cause the relaxation of compressive strain and it could be assumed that the strain reduced is proportional to the number of atoms diffused. Thus, the strain ε has a maximum value at the center of the grain, decays exponentially with distance x from the center point, and reaches zero at the grain boundary because of the much faster diffusion along grain boundaries. The strain at a point (x,y) inside the grain is given by Equation (6).

(6) $$\varepsilon \left(x,y\right)=\varepsilon_{\max}\left[1-\exp\left(-\frac{L_{a}/2-x}{L}\right)-\exp\left(-\frac{L_{a}/2-y}{L}\right)\right],$$

where ε_max_ is the strain at the center of the grain. The average strain $\bar{\varepsilon }$ inside a grain can be calculated by Equation (7).

(7) $$\bar{\varepsilon }=\frac{1}{{\left(L_{a}/2\right)}^{2}}\int_{0}^{L_{a}/2}\int_{0}^{L_{a}/2}\varepsilon \left(x,y\right)\mathrm{dx}\mathrm{dy}.$$

Integrating Equation (7), the average strain $\bar{\varepsilon }$ is given by Equation (8).

(8) $$\bar{\varepsilon }=\varepsilon_{\max}\left\{1-\frac{4L}{L_{a}}\left[1-\exp\left(-\frac{L_{a}/2}{L}\right)\right]\right\}\approx \varepsilon_{\max}\left(1-\frac{4L}{L_{a}}\right).$$

Equation (8) shows that the relation of the average strain $\bar{\varepsilon }$ with 1/L_a_ is linear, thus the relation of 2D-band frequency shift Δω_2D_ with 1/L_a_ is also linear according to Equation (2), which explains the Raman results in Figure 4a. The intercept of the fitting line for 2D-band in Figure 4a is 2714 cm^−1^, which corresponds to a compressive strain of 0.47%. The value of 0.47% well matches with the results in Ref. [8]. 

## 4. Conclusions

Raman spectra of CVD-EG show that abundant boundary-like defects exist in the graphene film and a significant blue-shift of 2D-band position is observed. The frequency of the 2D-band decreases linearly as 1/L_a_ increases and the G-band frequency increases linearly as 1/L_a_ increases. The opposite variation direction of G and 2D bands results from the presence of grain boundaries in graphene with small crystallite size. The downshift of 2D-band is caused by strain relaxation due to grain boundary diffusion.
